# Therapeutic drug monitoring of voriconazole: a case report of multiple drug interactions in a patient with an increased CYP2C19 activity

**DOI:** 10.1186/1742-6405-11-25

**Published:** 2014-08-04

**Authors:** Yassine Bouatou, Caroline Flora Samer, Kuntheavy Roseline Ing Lorenzini, Youssef Daali, Samira Daou, Marc Fathi, Michela Rebsamen, Jules Desmeules, Alexandra Calmy, Monica Escher

**Affiliations:** 1 Division of Clinical Pharmacology and Toxicology, Geneva University Hospitals, Geneva, Switzerland; 2 Division of Nephrology, Geneva University Hospitals, Geneva, Switzerland; 3 Swiss Centre for Applied Human Toxicology (SCAHT), Geneva, Switzerland; 4 Division of Infectious Diseases, Geneva University Hospitals, Geneva, Switzerland; 5 Division of Laboratory Medicine, Geneva University Hospitals, Geneva, Switzerland

**Keywords:** Drug interaction, Pharmacogenetics, Individualization, Therapeutic drug monitoring, CYP, antifungal

## Abstract

**Background:**

Voriconazole is metabolized by cytochrome P450 (CYP) 2C19 and CYP 3A4. Drug-drug interactions and genetic polymorphisms modulate their activities.

**Case presentation:**

A 35-year old African female patient with resistant HIV and a cerebral mass of unknown origin was treated with voriconazole for a suspicion of disseminated Aspergillosis infection. Voriconazole trough concentrations (C_0_) were within target range while the patient was under esomeprazole, a CYP2C19 inhibitor. Phenotyping showed decreased CYP2C19 activity, whereas genotyping showed a variant allele associated with increased enzyme activity. The patient was switched to ranitidine because of the introduction of atazanavir. CYP3A4 inhibition by atazanavir combined with uninhibited CYP2C19 activity resulted in subtherapeutic voriconazole C_0._ The reintroduction of esomeprazole allowed restoring voriconazole C_0_ back to target range.

**Conclusion:**

The integration of drug-drug interactions and pharmacogenetics data is crucial to interpret drug concentrations correctly, thus preventing suboptimal exposure to voriconazole.

## Case report

Voriconazole is used as an antifungal agent in the treatment of suspected or known Aspergillosis infection. [1] Voriconazole is mainly metabolized by cytochrome P450 (CYP) 2C19 and 3A4, [2] and its clearance is modulated by drug – drug interactions (DDI) [3,4] and genetic polymorphisms [5-7]. We report a case of multiple DDI in a patient with an increased CYP2C19 activity that led to variations in voriconazole trough concentrations (C_0_). Voriconazole C_0_ reached and stayed within the targeted range with the use of inhibitors of CYP2C19 and CYP3A4.

A 35-year old Cameroonian female was diagnosed with AIDS (CDC stage C3) in May 2012. A duodenal histoplasmosis and cryptococcosis infection were treated from June 2012 with a 3-week course of amphotericin B – flucytosine followed by oral voriconazole 100 mg bid. A combined antiretroviral regimen (cART) was initiated (emtricitabine, tenofovir and raltegravir). As she developed a single large intracranial mass, voriconazole was given intravenously and doses were increased up to 4 mg/kg/12 h. A cerebral biopsy showed aspecific inflammatory infiltrates with-T lymphocytes, negative gram stain and broad range PCR. As the lesion was associated with a voluminous edema, she was started on IV dexamethasone (3 mg tid) progressively tapered over 1 month. The proton pump inhibitor (PPI) esomeprazole 40 mg bid was started because of severe epigastralgia associated with duodenal histoplasmosis.

**Figure 1:**
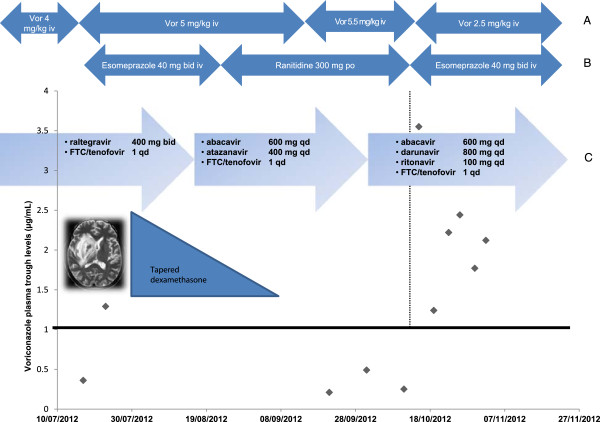
**Time course of patient treatment and therapeutic drug monitoring results.** Time course of **(A)** voriconazole i. v. dosage administration, **(B)** comedication with esomeprazole or ranitidine and **(C)** associated antiretroviral therapy. Voriconazole plasma trough concentrations. qd: once a day; b.i.d.: Twice a day.

Voriconazole first C_0_ was subtherapeutic (<1.0 μg/mL; range 1.0 - 5.0 μg/mL [3]) (Figure 1). The patient was genotyped for *CYP2C19*. It came heterozygous for the variant allele CYP2C19*17 (c.-860C > T; rs12248560) that is associated with an increased enzyme activity. Phenotyping with 2 mg omeprazole, while on CYP2C19 inhibitor esomeprazole, showed decreased CYP2C19 activity (omeprazole metabolic ratio =10.5; cut-off = 5). At that time voriconazole C_0_ was within the therapeutic range.

Due to several HIV resistance mutations and hepatitis B status, cART was modified with the addition of abacavir in order to increase cerebral penetration of unboosted atazanavir together with maintenance of emtricitabine/tenofovir. Atazanavir absorption depends on gastric acidity. The patient was switched from esomeprazole to the histamine-2 receptor antagonist ranitidine, which is less potent in suppressing intragastric pH.

Several voriconazole C_0_ were then measured below the therapeutic range despite substantial increased dosing (Figure 1). Darunavir - ritonavir regimen was introduced because of therapeutic failure and atazanavir was stopped. The patient presented with new onset epigastralgia and anemia. Ranitidine was replaced by esomeprazole 40 mg IV bid. Three days later, voriconazole C_0_ increased 14-fold and the voriconazole dose was reduced by 50% (2.5 mg/kg bid). There were no other medication changes. Subsequent voriconazole C_0_ stayed within the therapeutic range. However the patient’s condition deteriorated as she developed progressive multifocal leukoencephalopathy. She eventually died four months later.

CYP2C19 activity is modulated both by genetic polymorphisms and drug – drug interactions. Our patient was heterozygous for CYP2C19*17. Its frequency is approximately 21% in Caucasians, 16% in African-Americans, and 3% in Asians [8]. This allele is associated with increased enzymatic activity and may decrease voriconazole exposure compared to homozygous wild type extensive metabolizers [7,9]. CYP2C19 phenotype was performed [10] under high doses of esomeprazole, a CYP2C19 inhibitor. It explains the reduced enzymatic activity despite the patient’s genotype. Voriconazole C_0_ was within the therapeutic range when associated with the proton pump inhibitor [11]. It was subtherapeutic when the patient received ranitidine, which has no influence on CYP activity and voriconazole concentrations [12]. Atazanavir is a CYP3A4 inhibitor [13]. Hence voriconazole clearance depended only on uninhibited CYP2C19. When atazanavir was stopped, boosted darunavir was introduced. Ritonavir is a strong CYP3A4 inhibitor and can lead to increased voriconazole concentration. [2] However repeated administration of ritonavir induces CYP2C19 metabolic activity and potentially causes a reduction in voriconazole exposure [14]. The magnitude of the effect depends on the dose administered. The net effect of low dose ritonavir and inhibition of CYP2C19 by high-dose esomeprazole is likely to be a decrease in enzyme metabolic activity.

After esomeprazole was reintroduced, the patient was taking both a CYP2C19 and a CYP 3A4 inhibitor, and voriconazole plasma levels reached therapeutic concentrations.

Double blockade of voriconazole metabolic pathways (CYP2C19 and CYP3A4) led to concentrations within the therapeutic range in a patient genetically susceptible to subtherapeutic voriconazole concentrations. This case illustrates how the integration of drug-drug interactions and pharmacogenetics can help interpret drug concentrations during therapeutic drug monitoring, and thus minimize the risk of suboptimal exposure to voriconazole. The “boosting strategy” is mainly used in the fields of HIV therapeutics with antiretroviral drugs such as ritonavir or cobicistat and its efficacy to overcome specific CYP genotypes has not been evaluated so far.

## Consent

Written informed consent was obtained from the patient for publication of this Case report and any accompanying images. A copy of the written consent is available for review by the Editor of this journal.

## Competing interests

Alexandra Calmy has received grants from Abbott, Janssen Cilag and Gilead; these grants are unrelated to the present study. The other authors declare that they have no conflicts of interests.

## Authors’ contributions

YB, ME, AC, CS, SD, JD managed the case, and were involved in the interpretation of the data. YB drafted the manuscript. ME, CS, AC, KIL, helped in drafting the manuscript. YD performed and interpreted the phenotype measurement for CYP2C19; Michela Rebsamen performed and interpreted *CYP2C19* genotyping. Marc Fathi did the therapeutic drug monitoring. All authors were involved in critically revising the manuscript. They all read and approved the final manuscript.
